# A novel P53/POMC/Gαs/SASH1 autoregulatory feedback loop activates mutated SASH1 to cause pathologic hyperpigmentation

**DOI:** 10.1111/jcmm.13022

**Published:** 2016-11-25

**Authors:** Ding'an Zhou, Zhiyun Wei, Zhongshu Kuang, Huangchao Luo, Jiangshu Ma, Xing Zeng, Ke Wang, Beizhong Liu, Fang Gong, Jing Wang, Shanchuan Lei, Dongsheng Wang, Jiawei Zeng, Teng Wang, Yong He, Yongqiang Yuan, Hongying Dai, Lin He, Qinghe Xing

**Affiliations:** ^1^ Department of Laboratory MedicineYongchuan HospitalChongqing Medical UniversityChongqingChina; ^2^ Children's Hospital and Institutes of Biomedical SciencesFudan UniversityShanghaiChina; ^3^ Bio‐X InstituteKey Laboratory for the Genetics of Developmental and Neuropsychiatric Disorders (Ministry of Education)Shanghai Jiao Tong UniversityShanghaiChina; ^4^ Department of Laboratory MedicineThe Affiliated Hospital of North Sichuan Medical CollegeNanchongChina; ^5^ Dujiangyan People's HospitalCheng duSichuanChina

**Keywords:** SASH1, p53, DUH, hyperpigmentation

## Abstract

p53‐Transcriptional‐regulated proteins interact with a large number of other signal transduction pathways in the cell, and a number of positive and negative autoregulatory feedback loops act upon the p53 response. P53 directly controls the POMC/α‐MSH productions induced by ultraviolet (UV) and is associated with UV‐independent pathological pigmentation. When identifying the causative gene of dyschromatosis universalis hereditaria (DUH), we found three mutations encoding amino acid substitutions in the gene SAM and SH3 domain containing 1 (SASH1), and SASH1 was associated with guanine nucleotide‐binding protein subunit‐alpha isoforms short (Gαs). However, the pathological gene and pathological mechanism of DUH remain unknown for about 90 years. We demonstrate that SASH1 is physiologically induced by p53 upon UV stimulation and SASH and p53 is reciprocally induced at physiological and pathophysiological conditions. SASH1 is regulated by a novel p53/POMC/α‐MSH/Gαs/SASH1 cascade to mediate melanogenesis. A novel p53/POMC/Gαs/SASH1 autoregulatory positive feedback loop is regulated by SASH1 mutations to induce pathological hyperpigmentation phenotype. Our study demonstrates that a novel p53/POMC/Gαs/SASH1 autoregulatory positive feedback loop is regulated by SASH1 mutations to induce pathological hyperpigmentation phenotype.

## Introduction

The skin pigmentation is originated from the synthesis of melanin in the melanocytes, followed by distribution and transport of pigment granules to neighbouring keratinocytes 1. Variations in the coding region of the melanocortin‐1‐receptor (MC1R) are important for tanning and pigmentation in human beings. MC1R is a G protein‐coupled receptor (GPCR) that is preferentially expressed in epidermal melanocytes 2 and is activated by its ligand α‐melanocyte‐stimulating hormone (α‐MSH), a propigmentation hormone which is produced and secreted by both keratinocytes and melanocytes in the skin following UV. α‐melanocyte‐stimulating hormone and other bioactive peptides are cleavage products of pro‐opiomelanocortin (POMC), a multi‐component precursor for α‐MSH (melanotropic), ACTH (adrenocorticotropic) and the opioid peptide β‐endorphin. Normal synthesis of α‐MSH and ACTH is an important determinant of constitutive human pigmentation and the cutaneous response to UV 2.

Within melanocytes, MC1R regulates the amount and type of pigment production and is a major determinant of skin phototype, sensitivity to UV radiation‐induced damage and skin cancer risk 3. Upon ligand binding, GPCRs impart a signal to heterotrimeric G proteins, which are composed of α‐, β‐ and γ‐subunits, resulting in the detachment of the α‐subunit from the Gβγ subunit of G proteins. G proteins of the Gαs class directly catalyse the transformation of ATP to cAMP. cAMP is responsible for melanogenic actions of such ligands as α‐MSH, including the activation of tyrosinase in melanin biosynthesis 4.

The tumour‐suppressor protein p53, a transcriptional factor, has been documented to directly activate transcription of numerous genes such as those that control cell‐cycle, apoptosis and others. P53 directly mediates UV induction of POMC/MSH in skin and stimulates the POMC promoter in response to UV and is involved in UV‐independent pathologic pigmentation and could mimic the tanning response 1. In the skin, p53 function is critical for the retention of tissue integrity following UV irradiation 1. UV can exclusively induce dipyrimidine C to T substitutions that include CC to TT frameshift mutations in the p53 gene, which were found in the skin of UV‐irradiated mice months before tumour development 5. In addition to the above activities, p53 has been shown to be essential for the formation of ‘sunburn cells’, which are a hallmark of sunburns 5.

DUH is a clinically heterogeneous disorder that is characterized by generalized mottled pigmentation. DUH was initially described by Ichikawa and Hiraga in two generations of two families in 1933 6. We discovered similar Chinese DUH pedigrees with dyschromatosis symmetrica hereditaria (DSH) in 2003 with autosomal‐dominant DUH 7 and diagnosed as DUH rather than DSH subsequently. However, the pathological gene and pathological mechanism of DUH have not been further characterized since its first report in 1933.

SASH1 was originally described as a candidate tumour‐suppressor gene in the carcinomas of breast and colon and belongs to the previously described novel family of putative adapter and scaffold proteins that transfer signals from the ligand to the receptor 8, 9, 10. Our previous findings indicate that SASH1 binds to Gαs, the downstream molecule of α‐MSH/MC1R signalling cascade 11. Our previous study also showed that, in several DUH several affected individuals, hyperpigmented macules were showed to become more pronounced after strong UV exposure especially in summer 7, but no further mechanism was identified the reasons of photosensitivity 12. The importance of expression of p53/POMC/α‐MSH in UV‐photopigmentation response and UV‐independent hyperpigmentation has been elucidated 1. Moreover, less observations were reported to demonstrate that the variations in *SASH1* gene are associated with hyperpigmentation and how these variations cause hyperpigmentation.

Taken above, we hypothesise that a novel p53/POMC/α‐MSH/Gαs that SASH1 is involved in, to mediate UV‐photopigmentation response and pathological hyperpigmentation.

## Materials and methods

### PCR, sequencing and mutation analysis

Two Chinese families from the Henan and Yunnan provinces of China and one American family with typical features of DUH were recruited for this study. Three pedigrees with DUH showed an autosomal‐dominant inheritance pattern and were ascertained by experienced clinical dermatologists. The American family is a smaller pedigree, which could only provide three peripheral blood samples from affected individuals for study. This research was approved by the ethical review committees from the appropriate institutions. Genotyping was performed, and the two‐point LOD score was calculated as previously described 7. In total, 50 family members and 500 normal individuals (controls) participated in the study after providing informed consent. Samples of peripheral blood DNA were taken from all available family members. PCR and sequencing were performed as previously described 7. The sequencing was performed with an ABI BigDye Terminator Cycle Sequencing Kit (Applied Biosystems Inc, Foster City, CA, USA) on an ABI PRISM 3130 DNA Analyzer (Applied Biosystems), and data were analysed using sequence analysis software, version 3.4.1 (Applied Biosystems). Sequence data were compared with the SASH1 reference sequence (GenBank NM_015278.30) using Sequencher 4.10.1 (Gene Codes Corp, Ann Arbor, Michigan, USA). Nucleotide numbering reflects complementary DNA (cDNA) numbering, with +1 corresponding to the A of the ATG translation initiation codon in the reference sequence 7.

### Construction of SASH1, Gαs, POMC and p53 expression vectors

The construction protocol of recombined vector of wt and mutant SASH1‐PEGFP‐C3 and wt and mutant SASH1‐PBABE‐Flag‐puro was mainly referred to our previous study 11. To construct HA‐Pcna3.0‐p53, myc‐Pcdna3.0‐POMC and GFP‐Gαs‐Pegfp‐C3 vectors, PCRs of bacteria (obtained from Han jiahuai Lab, Xiamen University, Xiamen, China) containing the vector of full‐length CDS sequences of Gαs, p53 and POMC were performed with Phusion Hot Start High Fidelity Polymerase (New England Biolabs, Inc., Ipswich, Massachusetts, USA) or GXL Polymerase (Takara, Shimogyo‐ku, Kyoto, Japan), and the following primers were used: Gαs primers 5′‐ACGCGTCGACATGGGCTGCCTCGGGAAC‐3′ (forward, *Sal*I site included) and 5′‐CCGCTCGAG TTAGAGCAGCTCGTACTGACG‐3′ (reverse, *Xho*I site included); p53 primers 5′‐CGCGGATCCGCCACCACCATGGAGGAGCCGCAGTCAGATCCTA‐3′ (forward, *BamH*I site included) and 5′‐CCGCTCGAGTCAGTCTGAGTCAGGCCCTTCTGT (reverse, *Xho*I site included); POMC primers 5′‐CGCGGATCC ATGCCGAGATCGTGCTGC‐3′ (forward, *BamH*I site included) and 5′‐CCCAAGCTTT CACTCGCCCTTCTTGTAGGCGTTCTTGAT‐3′ (reverse, *Xho*I site included). Mammalian expression vectors (Invitrogen, Carlsbad, California, USA) *via* the relative restriction sites were sequenced.

### Cell culture and transfection

A375 cells, SK‐MEL‐28 cells and HEK‐293T cells were maintained as previously described 13. Normal human epithelial melanocytes (NHEMs, C‐12402; PromoCell, Germany) were cultured in M2 medium. A375, SK‐MEL‐28 and HEK‐293T cells were transfected using Lipofectamine 2000 (11668‐027; Invitrogen) as previously described 13, 14 or Entranster‐D (18668‐01; Engreen Biosystem Co., Ltd, New Zealand) or polyethyleneimine (PEI) prepared by ourselves. The transfected A375 and SK‐MEL‐28 cells were cultured in 1.5 μg/ml puromycin or 2.0 μg/ml G418 to select stable cell lines. HEK‐293T cells were transiently transfected with wild‐type and mutant SASH1‐pEGFP‐C3 or co‐transfected with wild‐type SASH1‐Pbabe‐Flag‐puro and Gαs‐Pegfp‐C3 vectors for immunoprecipitation experiments. NHEMs and HEK‐293 or HEK‐293T cells were transiently transfected with Pcdna3.0‐HA‐p53, Pcdna3.0‐myc‐POMC, Pegfp‐C3‐Gαs and wild‐type SASH1‐pEGFP‐C3 according to pairwise combination to analyse the expression of exogenous p53, POMC, Gαs and SASH1 using PEI prepared by ourselves or PromoFectin (PK‐CT‐2000‐MAC‐1; PromoCell, Heidelberg, Germany).

HEK‐293T cells were transfected with Gαs‐GFP, HA‐p53, myc‐POMC and GFP‐SASH1 vector and subsequently silenced by Gαs‐ and POMC‐specific siRNAs that were synthesized by Shanghai GenePharma Co., Ltd (Shanghai, China) using Entranster^™^‐R Transfection Reagent (18668‐06; Engreen Biosystem Co., Ltd). The sense and antisense strands of each siRNA for Gαs, POMC, GAPDH and the negative control are shown in Table S3.

### Pull‐down assay and nano‐flow LC‐MS/MS and bioinformatic analysis

The protocols for the pull‐down assay, nano‐flow LC‐MS/MS, database search and bioinformatic analysis for functional classification are mainly referred to our previous report 11.

### Immunoprecipitation and immunoblotting

HEK‐293T or HEK‐293 transfected cells and NHEMs were gently washed in PBS three times and then lysed for 20 min. using IP‐Western blot lysis buffer (P0013; Beyond Time BioScience and Technology company, Jiangshu, China) in the presence of a complete protease inhibitor cocktail, 1 μM sodium orthovanadate and 1 mM sodium fluoride per 10‐cm dish on ice. Cell lysates were transferred into 1.5‐ml microcentrifuge tubes. Extracts were centrifuged for 10 min. at 13400g. at 4°C. Then, 600 μl of supernatants was pre‐cleaned with 20 μl of Protein A/G PLUS‐Agarose (sc‐2003; Santa Cruz Biotechnology, Inc, California, USA) for 1 hr, immunoprecipitated using 6 μl of GFP‐Tag (7G9) mouse mAb (M20004, Shanghai Abmart, Inc., Shanghai, China) or 6 μl of DYKDDDDK‐Flag‐Tag mouse mAb (M20008; Shanghai Abmart, Inc.) or 6 μl of HA‐Tag mouse mAb (SG4110‐25; Shanghai Genomics, Shanghai, China) at 4°C for 10 hr and mixed with 20 μl of Protein A/G PLUS‐Agarose (sc‐2003, Santa Cruz Biotechnology, Inc.) at 4°C for 4 hr and assayed using co‐immunoprecipitation or immunoprecipitation. The immunoprecipitates were washed with PBS three times and subjected to SDS‐PAGE and Western blotting. The primary antibodies used in the Western blot analysis were GFP‐Tag mouse Ab (M20004, Shanghai Abmart, Inc.), Flag‐tag mouse mAb (M20008; Shanghai Genomics), anti‐Gαs rabbit polyclonal Ab (G7X105877; Gene Tex, Inc., Irvine, CA, USA), myc‐tag mAb (SG411‐30, Shanghai Genomics) and HA‐tag mouse mAb (SG4110‐25, Shanghai Genomics), SASH1 Rabbit mAb (A302‐265A‐1, Bethyl Laboratories, Inc., Montgomery, Texas, USA), DYKDDDDK‐Flag‐Tag mouse mAb (M20008; Shanghai Abmart, Inc.), TYRP1 (TA99) mouse mAb (Ab3312; Abcam, Cambridge, UK), Rab 27a mouse mAb (H0005873‐M01; Abnova, Taipei City, Taiwan), melanoma gp100 Rabbit mAb (ab137062; Abcam, Cambridge, UK), GAPDH mouse mAb (M20005; Shanghai Abmart, Inc.) and anti‐β‐tubulin mouse mAb (M20005M; Shanghai Abmart, Inc.). Immunoblotting was performed as previously described 15.

### Immunohistochemical and immunofluorescence staining, and melanin staining

#### Immunohistochemical staining

Written informed consent regarding tissue and data use for scientific purposes was obtained from all participating patients. Epithelial tissues from affected individuals with the Y551D SASH1 mutation from pedigree family I were fixed in 10% formalin at 4°C for 24 hr and then embedded in paraffin. Paraffin sections (5 μm) were incubated at 56°C overnight and then deparaffinized and rehydrated using xylene and an ethanol gradient. The sections were incubated with the SASH1 monoclonal antibody (A302‐265A‐1; Bethyl Laboratories, Inc.), Rabbit Anti‐ACTH (7‐23) antibody (bs‐004R; biosynthesis biotechnology Co., Ltd, Beijing, China), Mitf polyclonal antibody (BS1550; Bioworld Technology, Inc, Louis Park, MN, USA), HMB45 monoclonal antibody (sc59305; Santa Cruz Biotechnology, Inc.), TYRP1 (TA99) mouse mAb (Ab3312; Abcam), Rab 27a mouse mAb (H0005873‐M01; Abnova) and p53 monoclonal antibody (kit‐0010‐2; biosynthesis biotechnology Co., Ltd) as well as horseradish peroxidase‐linked anti‐rabbit and antimouse universal secondary antibodies or FITC. Finally, sections were counterstained with haematoxylin and photographed under the positive position microscope BX51.

#### Immunofluorescence (IF) and confocal microscopy

Wild‐type or mutant SASH1‐A375 stable cells were plated in 6‐well chamber slides and incubated at 37°C for at least 48 hr. Indirect immunofluorescence analysis was performed on A375 cells expressing wild‐type and mutant SASH1 (s) in 6‐well chamber slides to assess SASH1 localization. IF was performed as described previously using the following antibodies: SASH1 Rabbit mAb (A302‐265A‐1; Bethyl Laboratories, Inc.) and DYKDDDDK‐Flag mouse mAb (M20008; Shanghai Genomics) 11.

#### Melanin staining

Paraffin sections (5 μm) from epithelial tissues were incubated in an 80°C baking oven for 30 min and then kept at room temperature for 15 min. Melanin staining was performed according to the manufacturer's protocol (GMS80023.3; GENMED SCIENTIFICS INC., Shanghai, China) and observed under a light microscope.

### Quantitative real‐time RT‐PCR

The total RNA from the different groups of SK‐MEL‐28 cells was isolated using TRIzol Reagent (Invitrogen). Reverse transcription was carried out according to the manufacturer's protocol for the PrimeScript^™^ RT Reagent Kit (DRR037A; Takara) or PrimeScript RT reagent using the gDNA Eraser Kit (DRR047A; Takara) for qRT‐PCR. The sense and antisense primer sequences for SASH1, TYRP1, Pmel17, Rab27a, Gαs, POMC and GAPDH are presented in Table S3. The PCR products were confirmed by agarose gel electrophoresis. Real‐time PCR was performed using the Applied Biosystems 7500 System with SYBR Premix Ex Taq^™^ (DRR041A; Takara). The quantity of each mRNA was normalized to that of GAPDH mRNA.

### UV exposure

The human foreskin tissues from a 14‐year‐old boy were exposed for enough time under an UV phototherapy instrument (NBUVB SS‐05; Sigma‐Aldrich, St. Louis, Missouri, USA) to reach the expected UV intensity, then fixed in 10% formalin and embedded in paraffin for immunohistochemistry analyses. We conformed to the guidelines of the World Medical Assembly (Declaration of Helsinki) to acquire the human foreskin tissues.

In the case of *in vitro* UV experiments which mainly referred to the protocol of our institute 16, HEK‐293T cells and NHEMs transiently transfected with myc‐POMC were cultured to approximately 70–80% confluence in 6‐cm‐diameter dishes and were irradiated with 100 mJ/cm^2^ UVC delivered *via* a HL‐2000 HybriLinker with a 254‐nm wavelength (Upvon) and followed by the indicated recovery time. Finally, cells were harvested to detect proteins’ levels using immunoblot.

### Electrophoretic mobility shift assay

Three probes binding with/without biotin, which targeted SASH1 promoter, were synthesized. The sequence of probes was as follows: probe 1# 5′‐GCCCAAGCTT TCACACTTGTTT‐3′, probe 2# 5′‐CCAAGACTTGCTAGAAGGAACGAGTCG‐ 3′, probe 3#5′‐CGTGGCCACCTAGACCCGAGGTG‐3′. Electrophoretic mobility shift assay was performed as described as the protocol provided with LightShift^®^ Chemiluminescent EMSA Kit (20148; Thermo Scientific, Pierce Biotechnology, Rockford, USA).

### Statistical analysis

The data are presented as mean ± standard error of the mean (S.E.M.)s. These data were first analysed using the homogeneity of variance test and followed by the change of variable test. Statistical significance was determined by a one‐factor analysis of variance (anova) with LSD correction on SPSS version 16.0 (IBM (International Business Machine)) to generate the required *P*‐values. Cartograms were plotted using GRAPHPAD PRISM 5 (GraphPad Software, Inc. La Jolla, CA, USA) 5.

## Results

### Mutations in *SASH1* in DUH‐affected individuals result in the up‐regulation of SASH1 *in vitro* and *in vivo*

**Figure 1:**
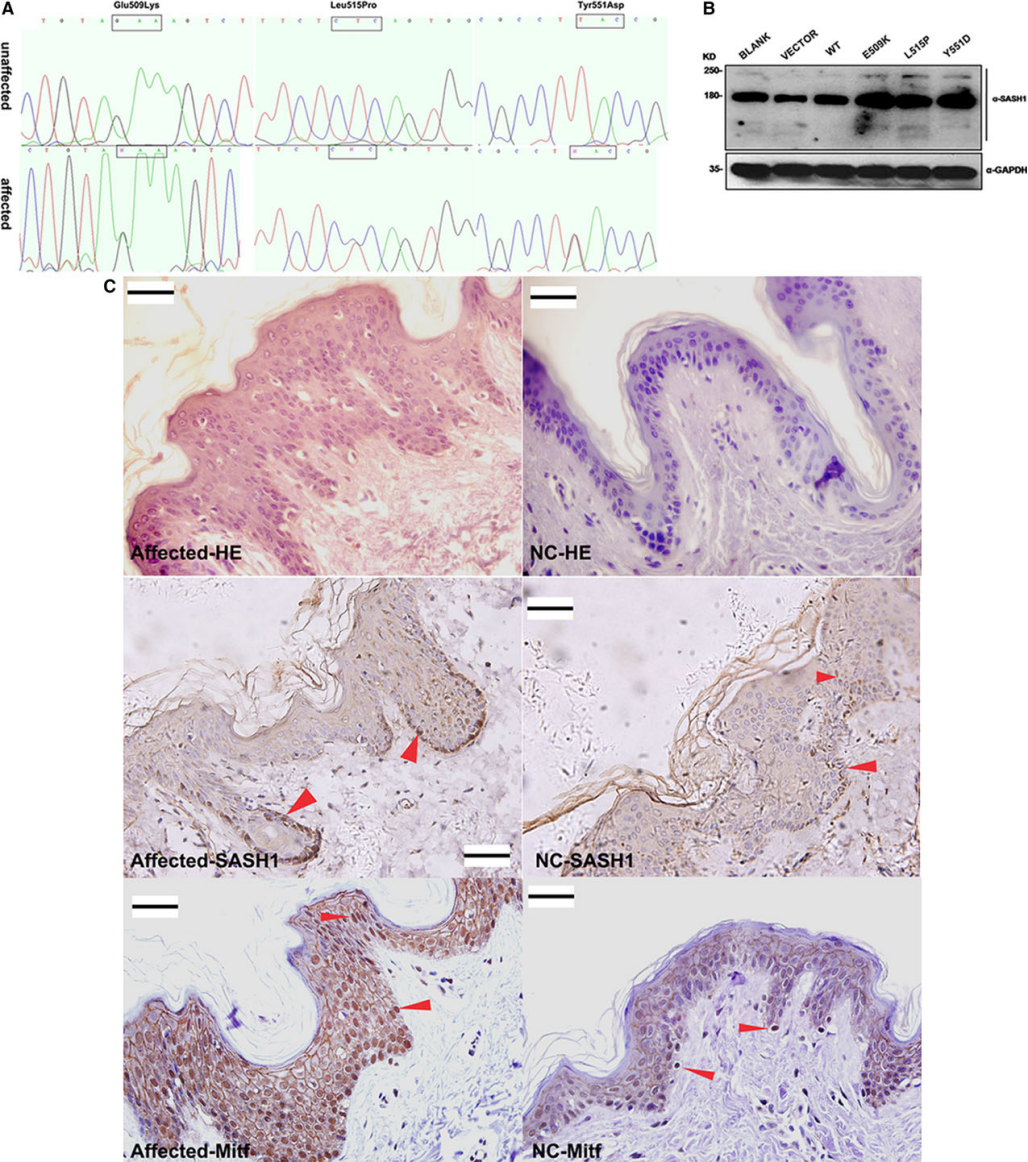
Mutations in SASH1 increase SASH1 expression *in vitro* and *in vivo*. (**A**) Mutation sites in the SASH1 gene in three families with DUH. (**B**) Western blotting demonstrated the differential and increased expression of mutant SASH1 proteins compared to that of wild‐type SASH1 in different A375 cells. (**C**) HE staining, SASH1 and Mitf immunohistochemical analysis of the epidermal tissues from the Y551D‐mutation DUH‐affected individuals and normal controls. Heterogeneous expression of the SASH1 protein was observed in all of the epithelial layers in the epidermal tissues from the Y551D‐mutation DUH‐affected individuals as compared with that of normal controls (NC). Heterogeneous distribution of melanocytes was detected in the epithelial layers of DUH‐affected individuals using the melanocyte marker Mitf as compared with that of normal controls. 40× magnification. Scale bar = 20 μm. Red arrows denote the representative positive cells of SASH1 and Mitf.

We have located the gene that is responsible for DUH is localized to chromosome 6q24.2‐q25.2 7. The 10.2‐Mb region on chromosome 6 (6q24.2‐q25.2) that is flanked by the markers D6S1703 and D6S1708 contained more than 50 candidate genes. We screened selected genes in this region for possible pathological mutations by directly sequencing the PCR products of exons that were amplified from genomic DNA of affected, unaffected and control individuals. We sequenced 50 candidate genes and found three heterozygous mutations encoding amino acid substitutions in SAM and SH3 domain containing I (SASH1) in the probands in each of the two non‐consanguineous Chinese DUH‐affected families (families I and II) and in one non‐consanguineous American DUH‐affected family (family III). SASH1 point mutations were found in the three pedigrees. These mutations were as follows: a T→G substitution at nucleotide 2126 in exon 14 in family I, a T→C substitution at nucleotide 2019 in exon 13 in family II and a G→A substitution at position 2000 in exon 13 in family III. These three nucleotide changes cause non‐conservative missense mutations in the SASH1 gene, resulting in the following amino acid substitutions: Tyr to Asp at codon 551 (TAC→GAC), designated as Y551D; Leu to Pro at codon 515 (CTC→CCC), designated as L515P; and Glu to Lys at codon 509 (GAA→AAA), designated as E509K (Fig. 1A). These sequence changes were confirmed in all of the affected family members but were not observed in unaffected family members, correlating the presence of the mutations with the presence of the phenotype. The mutations were not observed in any of the 500 normal controls or in any of the current databases, including the HapMap database. Therefore, these mutations are unlikely to be common single nucleotide polymorphisms (SNPs) 7.

When *SASH1* mutants were stably expressed in A375 cells, they significantly up‐regulated SASH1 (Fig. 1B). Immunoblotting demonstrated that SASH1 was up‐regulated in A375 cells stably expressing either wild‐type (WT‐A375 cells) or mutant SASH1 (mutant‐A375 cells, including E509K‐A375 cells, L515P‐A375 cells and Y551D‐A375 cells), compared to the expression of endogenous SASH1 in A375 cells expressing the pBABE‐puro empty vector (VECTOR‐A375 cells) or A375 cells without any transfected vector (BLANK‐A375 cells) (Fig. 1B). To verify the stability of SASH1 proteins, HEK‐293T cells stably expressing wild‐type or mutant SASH1 were treated with 20 μg/ml of the protein synthesis inhibitor cycloheximide (CHX) for the indicated times to assess the half‐life of SASH1. The protein levels of SASH1 decreased in a time‐course‐dependent manner in response to CHX treatment. Wild‐type SASH1 levels decreased with a half‐life of approximately 4 hr. However, with CHX treatment for 6 hr or longer, CHX began to degrade mutant SASH1 proteins. Therefore, the three mutant SASH1 proteins were more stable than the wild‐type, supporting the above observation that SASH1 mutants are expressed at higher levels than the wild‐type (Fig. S1A and B). Endogenous SASH1 was an unstable protein with a half‐life of approximately 3 hr (Fig. S1C).

We characterized the subcellular localization of SASH1 in A375 cells and skin epithelial layers. The endogenous SASH1 protein in VECTOR‐A375 cells and the skin epithelial layers from normal controls demonstrated a homogeneous pattern of expression (Fig. 1C and Fig. S2‐a4). However, in WT‐A375 cells and mutant‐A375 cells, activated SASH1 (through either the overexpression or mutation of SASH1) showed a pattern of heterogeneous expression (Fig. S2‐b4 to Fig. S2‐e4). The heterogeneous pattern of SASH1 *in vitro* was also observed *in vivo* (Fig. 1C). In addition, most of the SASH1‐positive cells were melanocytes that were nucleic positive for Mitf, a melanocyte marker, and demonstrated a heterogeneous distribution of melanocytes in the epithelial tissues of DUH‐affected individuals as compared with those of unaffected individuals. Some cytoplasm‐positive staining of Mitf is false positive (Fig. 1C). The phenomenon that melanocytes or SASH1‐positive epithelial cells localized not only to the basal layers but also to the suprabasal layers of the affected epidermal tissue is coincide with our previous conclusion that SASH1 mutations promote melanocyte migration 11.

### SASH1 is associated with Gαs and induced by the canonical p53/POMC/Gαs cascade

**Figure 2:**
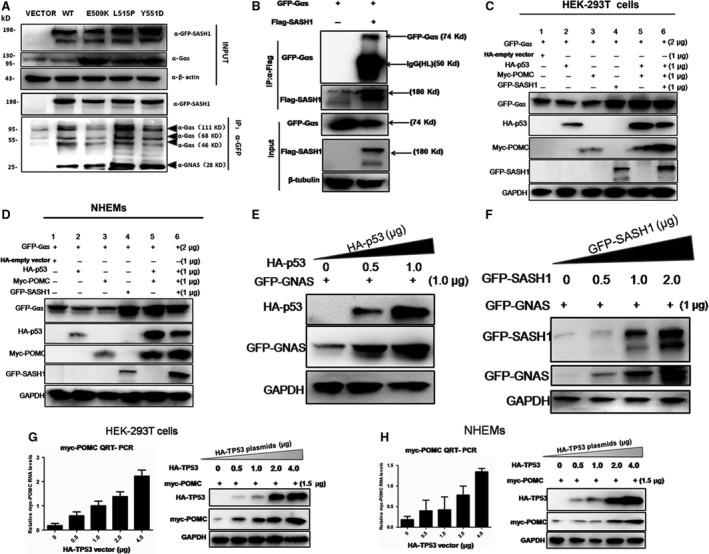
Gαs interacts with SASH1 and is a pivotal downstream of p53/POMC cascade. (**A**) The associations between GFP‐SASH1 and endogenous Gαs were identified by immunoprecipitate‐Western blot (IP‐WB) analysis in HEK‐293T cells. HEK‐293T cells were transfected with the pEGFP‐C3‐SASH1 vectors. At 24hr post‐transfection, GFP‐SASH1 was immunoprecipitated (IP), and the associated GFP‐SASH1 was detected by Western blot analysis using an anti‐GFP antibody. Different sizes of Gαs bands were observed, at 28, 46, 68 and 111 kD, which may be caused by post‐translational modifications (PTMs). (**B**) GFP‐Gαs is associated with Flag‐SASH1. HEK‐293T cells were co‐transfected with the pEGFP‐C3‐Gαs and pBABE‐puro‐Flag‐SASH1 vectors. At 36hr post‐transfection, Flag‐SASH1 was immunoprecipitated, and the associated GFP‐Gαs was detected by Western blot analysis using an anti‐GFP antibody. (**C**) and (**D**) P53, POMC and SASH1 is necessary for the activation of Gαs. HEK‐293 cells and NHEMs were transfected with HA‐p53, myc‐POMC and GFP‐SASH1, respectively, according to different manners of combination. After 36 hr after transfection, two normal cells were lysed and subjected to immunoblotting with GAPDH as loading control. (**E**) Exogenous Gαs is induced by p53. HA‐p53 and GFP‐Gαs were introduced into HEK‐293 cells. After 36 hr after transfection, cells were lysed and subjected to immunoblotting. Exogenous Gαs was activated by gradually increased amounts of exogenous p53(HA‐p53). (**F**) Exogenous Gαs is induced by SASH1. GFP‐Gαs and GFP‐SASH1 were introduced into HEK‐293T cells. Exogenous Gαs was induced by gradually increased doses of exogenous SASH1. (**G**) and (**H**) Exogenous p53 (HA‐p53) overexpression induces exogenous POMC(myc‐POMC) expression in a dose‐dependent manner in HEK‐293T cells and NHEMs. Different dose of HA‐p53 vector and a certain amounts of myc‐POMC vector were transfected into HEK‐293T cell for expression. Exogenous POMC RNA levels were measured by quantitative RT‐PCR and normalized to GAPDH. Results of RNA levels are expressed as the mean of the experiment carried out in triplicate ± the S.D. The expression of HA‐p53 and myc–POMC was analysed by Western blot as GAPDH as loading control.

The functional domains of SASH1 (SAM and SH3) suggest that this protein plays a role in a signalling pathway as a signalling molecule adapter or as an associated scaffolding protein 8, 9. Therefore, we performed a pull‐down assay and a mass spectrometry analysis to investigate which signalling pathways are activated by SASH1. The pull‐down assay and LC‐MS/MS analysis demonstrated that SASH1 interacts with Gαs and CALM, both of which are important in melanogenesis process (Table S1) in WT‐A375 cells. Gαs is a key element of the classical signal transduction pathway linking receptor‐ligand interactions with the activation of adenylyl cyclase and a variety of cellular responses 17. To investigate the associations between SASH1 and Gαs, HEK‐293T cells were co‐transfected with Flag‐SASH1 and GFP‐Gαs. Exogenous SASH1 was immunoprecipitated with both exogenous Gαs (GFP‐Gαs) and endogenous Gαs. Exogenous SASH1 immunoprecipitates had different observed band sizes of Gαs (Fig. 2A and C).

**Figure 3:**
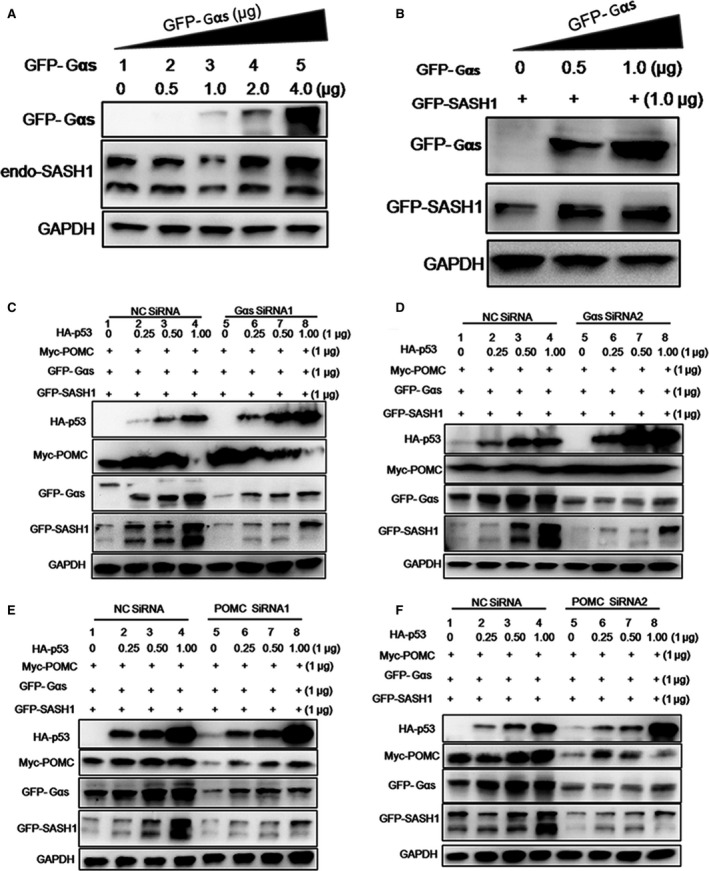
A novel p53/POMC/Gαs/SASH1 cascade regulates the expression of SASH1. (**A**) and (**B**) Endogenous or exogenous SASH1 is induced by Gαs. Gradually increasing amounts of exogenous Gαs (GFP‐Gαs) and exogenous SASH1 (GFP‐SASH1) or only different doses of exogenous Gαs were transfected into HEK‐293T cells. The expression of endogenous or exogenous SASH1 was analysed by immunoblotting along with GAPDH as loading control. (**C**) and (**D**) Gαs is necessary for the inducement of SASH1. After transfection with GFP‐Gαs, myc‐POMC and GFP‐SASH1 as well as increasing doses of HA‐p53 according to different combinations, two groups of HEK‐293 cells were subsequently introduced with two pairs of effective Gαs siRNAs and negative control (NC) siRNA. Protein levels were detected by immunoblot. (**E**) and (**F**) POMC is necessary for the inducement of SASH1 and Gαs. After introduction into GFP‐Gαs, myc‐POMC and GFP‐SASH1 as well as increasing dose of HA‐p53 according to different manner of combinations, two groups of HEK‐293 cells were subsequently silenced with two pairs of effective POMC siRNAs and NC siRNA.

Gαs mediates cAMP production in melanocytes which is stimulated by α‐MSH and melanocortins 18, and our study here shows that Gαs is associated with SASH1. Hence, we examine whether Gαs is required for the induction of SASH1 and how Gαs mediates SASH1 expression, we introduced exogenous p53, POMC, Gαs and SASH1 gene into HEK‐293T and NHEMs to assess the effects of p53 and POMC on Gαs. Exogenous Gαs was induced in the co‐existence of exogenous p53 and POMC (Fig. 2C lane 5 and Fig. 2D lane 5), and both inducements of exogenous Gαs and exogenous SASH1 were observed in the co‐existence of exogenous p53 and POMC in two types of normal cells (Fig. 2C lane 6 and Fig. 2D lane 6). Meanwhile, in the presence of GFP‐SASH1, GFP‐Gαs was also induced (Fig. 2C lane 4 and Fig. 2D lane 4), which indicated that SASH1 is necessary for the activation of GFP‐Gαs. And immunoblot showed that Gαs was identified to be induced by exogenous p53 and SASH1 (Fig. 2E and F). Our results also demonstrated that POMC was mediated by p53 in HEK‐293T and melanocytes were consistent with previous conclusions 1 (Fig. 2G and H). Conversely, endogenous SASH1 and exogenous SASH1 were induced by Gαs (Fig. 3A and B).

To confirm the fact that POMC, p53 and Gαs are necessary for the inducement of SASH1, exogenous POMC, p53, Gαs and SASH1 were transfected into HEK‐293T cells and followed by silence of Gαs and POMC by two specific pairs of siRNA, respectively. As identified in HEK‐293 cells, knockdown of Gαs gene directly induced significant reduction in SASH1 (Fig. 3C and D). Silencing of POMC resulted in the down‐regulation of Gαs and SASH1 (Fig. 3E and F).Taken above, it is believed that Gαs serves as a pivotal downstream of p53/POMC cascade and SASH1 is regulated by a novel p53/POMC/Gαs cascade.

### SASH1 is physiologically induced by p53 upon UV stimulation

**Figure 4:**
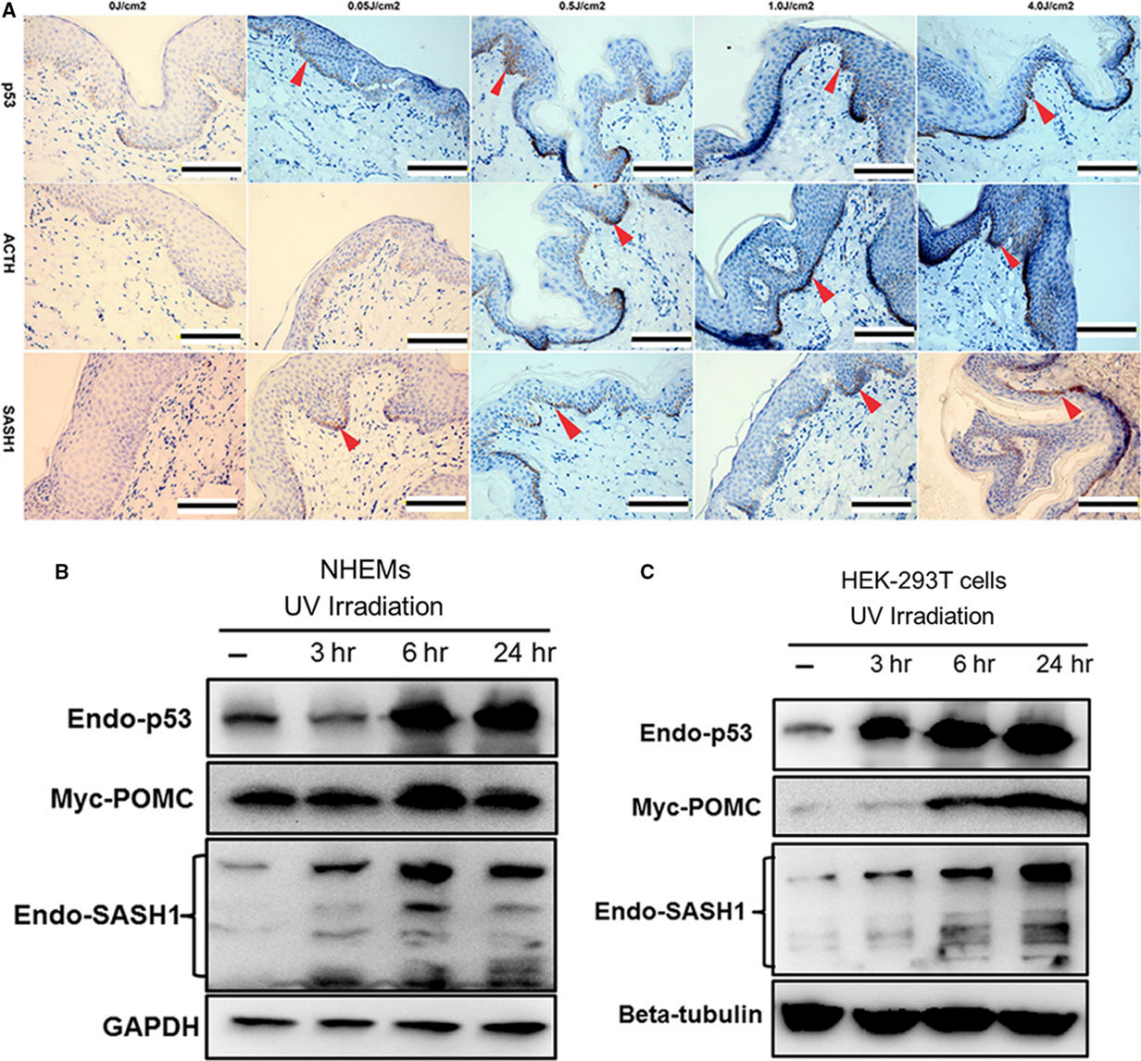
Upon UV irradiation, SASH1 is induced physiologically by p53. (**A**) Immunohistochemical staining of p53, POMC and SASH1 in human foreskin with or without UV irradiation showed that the p53 activation by UV‐induced up‐regulation of POMC and SASH1. The human foreskin tissues from a 14‐year‐old boy were exposed at different doses of UV intensity, then fixed in 10% formalin and embedded in paraffin for immunohistochemistry analysis. Scale bar: 20 μm. Red arrows indicate the representative positive cells of p53, ACTH and SASH1. (**B**) and (**C**) NHEMs and HEK‐293T cells transfected with exogenous POMC(myc‐POMC) were irradiated with UV irradiation (100 mJ/cm2) and recovered for the indicated times. Proteins were collected at different time‐points after irradiation as indicated. Endogenous p53, endogenous SASH1 and exogenous POMC protein levels were detected by Western blot. GAPDH or beta‐tubulin was used as a loading control.

To verify that SASH1 is induced physiologically by p53, discarded normal human foreskin specimens were exposed to gradually increased dose of UV and stained for the histological analyses of p53, ACTH/POMC and SASH1. Immunohistochemical (IHC) analyses revealed p53 is rapidly induced in basal layers at the 0.5J/cm^2^ dose of UV irradiation. The rapid induction of SASH1 and POMC/ACTH at 1.0J/cm^2^ dose of UV irradiation in melanocytes is followed by p53 up‐regulation (Fig. 4A). Previous reports had suggested that the up‐regulation of POMC gene is induced at both protein and mRNA levels following UV irradiation of skin 19, 20. Followed the previous descriptions 1, a 100 J/m^2^ UVB dose was administered in this experiment. This dose is equivalent to the standard erythema dose (SED), which is commonly used as a measure of sunlight 21. So HEK‐293T cells and NHEMs were transfected with exogenous POMC and followed by UV irradiation, both endogenous p53 and SASH1 protein levels were assessed by immunoblot. UV markedly induced expression of exogenous POMC and endogenous SASH1 by 6 hr, and p53 induction was already maximal by 3 hr, which is consistent with its known stabilization by UV in NHEMs. At 24 hr, the levels of POMC, p53 and SASH1 protein were maximally induced by UV in NHEMs (Fig. 4B). Similar inducement of exogenous POMC and endogenous p53 and SASH1 by UV irradiation was observed in HEK‐293T cells (Fig. 4C). Hence, we believe that not only POMC but also SASH1 acts as a novel downstream partner which is responsive to the induction of p53 by UV irradiation.

### Reciprocal induction between p53 and SASH1 is induced in normal cells

**Figure 5:**
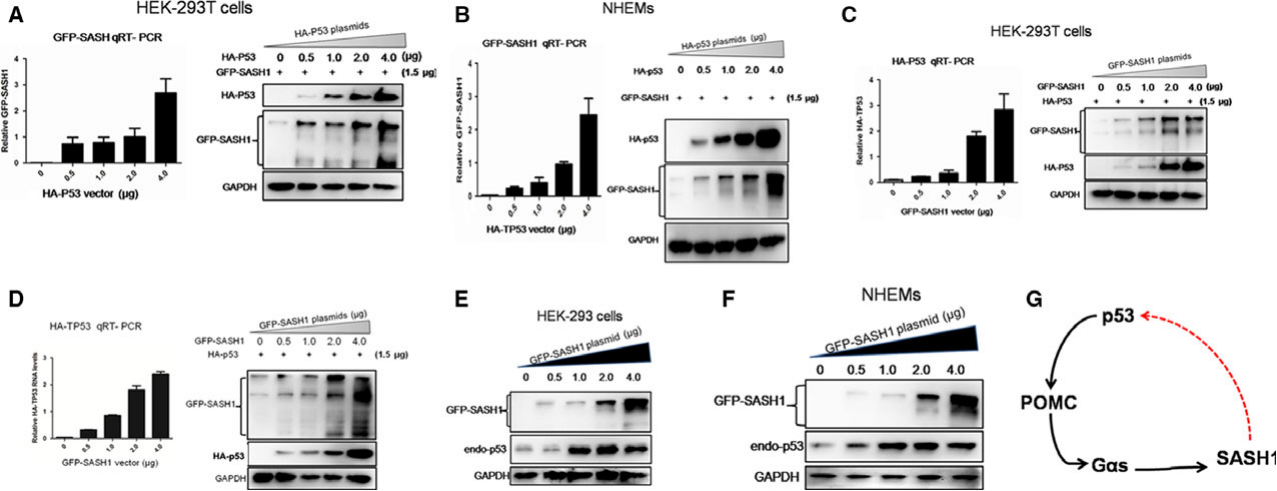
Reciprocal induction between p53 and SASH1 is induced in normal cells. (**A**) and (**B**) Exogenous p53 (HA‐p53) triggers exogenous SASH1 expression in a dose‐dependent manner. HEK‐293T cells and NHEMs were transfected with different amounts of HA‐p53 plasmid as indicated. Exogenous SASH1 RNA levels were measured by quantitative RT‐PCR and normalized to GAPDH. Expression of exogenous p53 protein and SASH1 was analysed by Western blot along with GAPDH as a loading control. (**C**) and (**D**) Exogenous SASH1 promotes expression of exogenous p53. Different amounts of GFP‐SASH1 plasmid and a certain amount of exogenous p53 were transfected to HEK‐293T cells and NHEMs cells and plasmid. Increasing amounts of GFP‐SASH1 trigger expression of exogenous p53, as analysed by QRT‐PCR and Western blot. (**E**) and (**F**) Exogenous SASH1 promotes expression of endogenous p53. Different amounts of exogenous SASH1 were introduced to HEK‐293T cells and NHEMs. After 36‐hr transfection, cells were lysed and subjected to Western blot to analyse the expression of GFP‐SASH1 as GAPDH as loading control. Results are the representative of three independent results. (**G**) An autoregulatory p53/POMC/Gαs/SASH1 loop mediates reciprocal induction of p53 and SASH1. p53 after being activated by different types of stress triggers the expression of POMC, Gαs and SASH1 successively. The induced SASH1 by p53/POMC/Gαs cascade promotes the up‐regulation p53 in nucleus, then induced nucleic p53 conversely activates POMC/Gαs/SASH1 cascade, which consists an autoregulatory p53/POMC/Gαs/SASH1 loop.

To examine whether p53 is required for the induction of SASH1, we introduced exogenous p53 and POMC gene into HEK‐293T and NHEMs to assess the induction of p53 and POMC to SASH1. Exogenous SASH1 was induced by p53 in the presence of POMC (myc‐POMC) in NHEMs and HEK‐293T cells (Fig. S3). Exogenous SASH1 was induced by increasing amounts of exogenous p53 in two normal cells (Fig. 5A and B). Conversely, exogenous p53 was promoted by increasing amounts of exogenous SASH1 (Fig. 5C and D). We also identify the inducement of exogenous SASH1 to endogenous p53. As documented in Figure 5E and F in two types of normal cells, endogenous p53 was induced by increasing dose of exogenous SASH1.

As SASH1 is regulated by p53, we intend to explore whether there is a direct association between SASH1 and p53. As shown in Figure S4A and Figure S4B, HA‐p53 is not associated with GFP‐SASH. So, we examined the proximal 1‐kb promoter region of the SASH1 gene to search for consensus transcription‐factor‐binding elements that are conserved between human, rat and mouse. Among the various consensus elements found, p53 gene was noteworthy. A most possible p53‐binding site, sequences of which is ‘tgcccaagctttcacacttgttt’, was found in the SASH1 5′ flanking region about 550‐bp upstream of the transcription initiation site in human beings (Fig. S4C). Three probes were synthesized to detect the binding of p53 protein with SASH1 gene promoter. However, electrophoretic mobility shift assay (EMSA) confirmed that there was no p53 protein bind the promoter region of the SASH1 gene(Fig. S4D).

Taken above, we believe that SASH is regulated by the p53/POMC/α‐MSH/Gαs cascade and a novel autoregulatory loop was consisted of p53/POMC/α‐MSH/Gαs cascade and SASH1. The p53/POMC/α‐MSH/Gαs/SASH1 regulatory loop serves as an auto‐feedback circuit to regulate reciprocal induction between p53 and SASH1 (Fig. 5G).

### SASH1 mutations enhance expression of p53 and POMC

**Figure 6:**
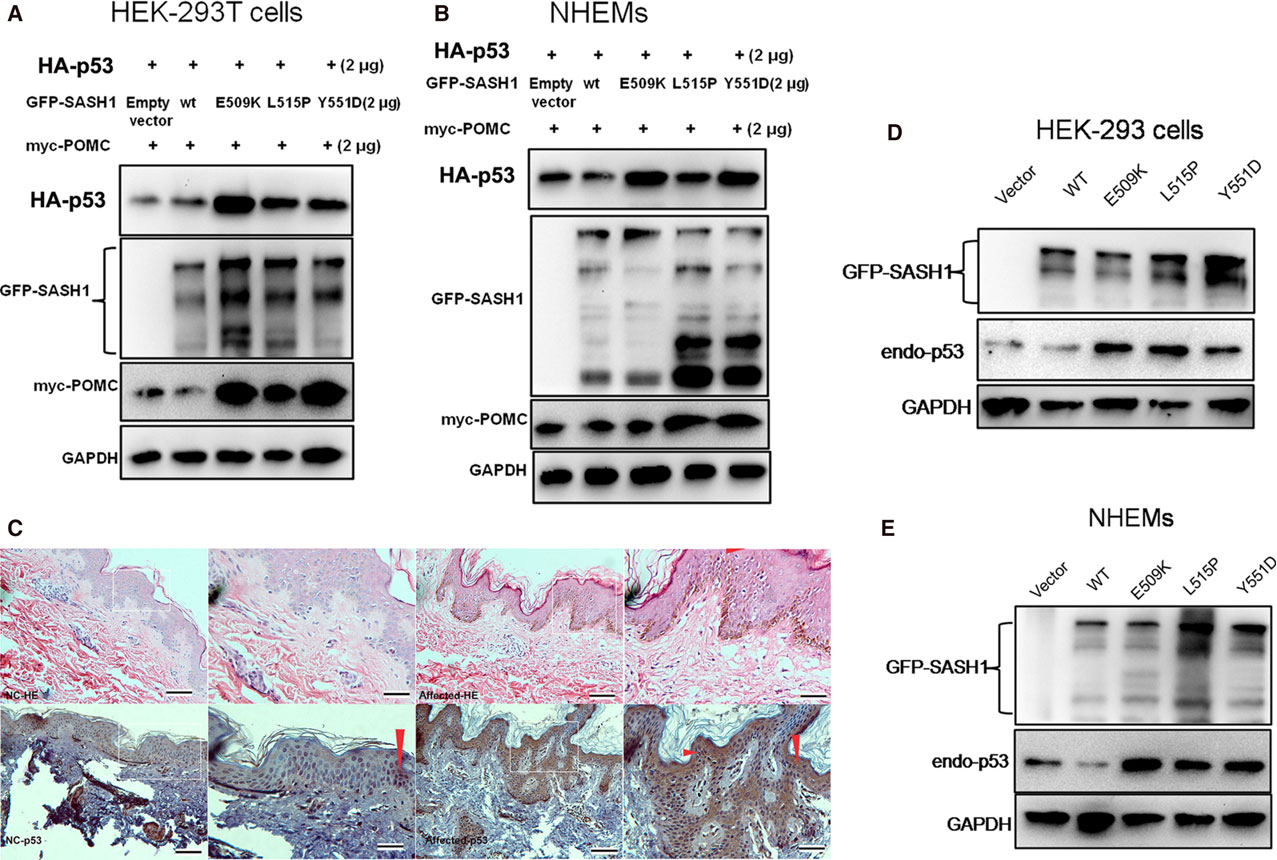
p53 is enhanced by SASH1 mutations and SASH1 mutations induce increased POMC. (**A**) and (**B**) SASH1 up‐regulation induced by SASH1 mutations promotes the expression of exogenous p53 (HA‐p53) and exogenous POMC(myc‐POMC) in HEK‐293T cells and NHEMs. HEK‐293T cells and NHEMs were transfected with wt and mutant SASH1, exogenous p53 and exogenous POMC. At 48hr post‐transfection, cells were lysed and protein levels were detected by immunoblot. (**C**) IHC analysis identified that more endogenous p53 was induced by Y551D‐SASH1 mutation and more p53‐positive epithelial cells was observed in the affected epithelial tissues. Affected epithelial tissues with Y551D SASH1 mutation from pedigree family I as well as normal epithelial tissues were fixed and embedded in paraffin for immunohistochemical analysis. Scale bar: 20 μm. Red arrows show the representative positive cells of p53. (**D**) and (**E**) Increased endogenous p53 was triggered by SASH1 mutations in HEK‐293 cells and NHEMs as analysed by Western blot.

The reasons of SASH1 mutations‐mediated up‐regulation itself are an enigma that plagues us for long term. So, wild‐type or mutant SASH1 (wt SASH1 or mut SASH1), p53 and POMC were introduced into HEK‐293T cells and NHEMs to test the effects of SASH1 mutations on p53 and POMC protein levels. As shown in Figure 6A and B, SASH1 mutations induce up‐regulation of p53 and POMC in two normal cell lines. We also assessed the effects of SASH1 mutations on endogenous p53 at protein level, and endogenous p53 up‐regulation is also induced by mutated SASH1 (Fig. 6D and E). To identify the fact that p53 is induced by SASH1 mutations in vivo, we performed immunostaining of p53 in the affected epithelial tissues with SASH1 Y551D mutation. IHC analysis demonstrated that SASH1 Y551D mutation induced more nucleic expression of p53 in epithelial tissues and more p53‐positive cells in affected epithelia layers (Fig. 6C).

All of these indicate that not only SASH1 is positively mediated by the p53/POMC/α‐MSH/Gαs/SASH1 autoregulatory loop but also SASH1 mutations serve more as molecular rheostats rather than an on/off switch to regulate this regulatory loop.

### SASH1 mutations trigger the expression of melanosomes matrix proteins *in vitro* and *in vivo*

As there is an autoregulatory loop between SASH1 and p53, the downstream regulatory players of SASH1 need to be investigated. The biosynthesis of melanin is sequestered within melanosomes, which are unique membrane‐enclosed structures in melanocytes. Melanosomes and their precursors can be classified into four stages of development according to their principal component of mature melanosomes 22. Rab27a plays a pivotal role in the transport of melanosomes to the dendrite tips of melanocytes, and mutations in Rab27a which impair melanosome transport cause the pigmentary dilution and immune deficiency found in several patients with Griscelli syndrome (GS) 23. Myosin VIIa and Rab27a, which co‐ordinately transport and constrain melanosomes within a region of filamentous actin, are particularly important for the motility and localization of melanosomes 24.

**Figure 7:**
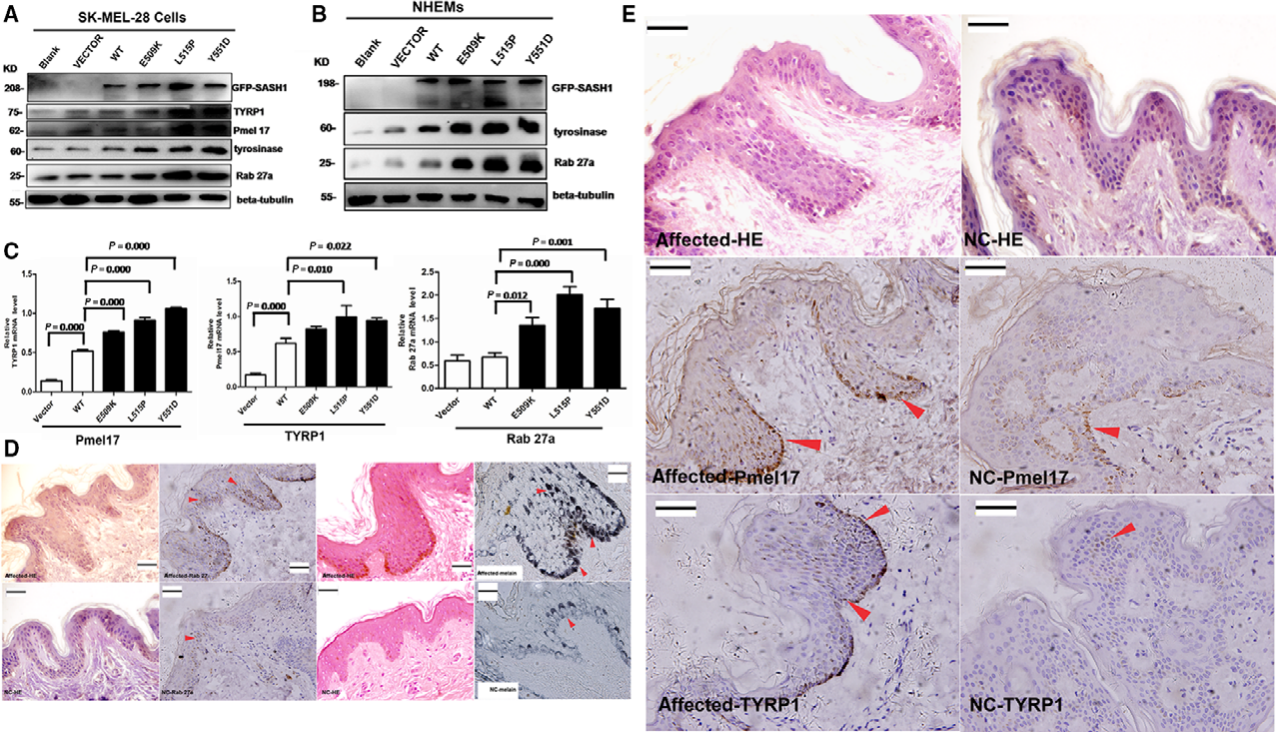
SASH1 mutations increase production of melanogenic components and induce heterogeneous distribution of melanin *in vivo*. (**A**) In stable SK‐MEL‐28 cells, SASH1 mutations also resulted in the up‐regulation of melanogenic components, including TYRP1, Pmel17, tyrosinase and Rab 27a. (**B**) The SASH1 gene (wt and mutant) was introduced into NHEMs to determine the effect of SASH1 mutations on melanogenic components. At 24hr post‐transfection, SASH1 also resulted in the up‐regulation of Rab 27a and tyrosinase. (**C**) QRT‐PCR demonstrated that SASH1 mutations resulted in the up‐regulation of Pmel17, TYRP1 and Rab 27a in stable SK‐MEL‐28 cells (*n* = 4, mean ± standard error). (**D**) IHC demonstrated that Rab 27a and melanin were distributed heterogeneously in the epithelial layers of the affected individuals. (**E**) Heterogeneous expression of Pmel17 and TYRP1 was observed in all of the epithelial layers of the epidermal tissues from the DUH‐affected individuals. Pmel17, TYRP1 and Rab 27a: 40× magnification, bar = 20 μm; melanin: 100× magnification. Scale bar:20 μm. Red arrows indicate the representative positive cells of Rab 27a, Pmel17, TYRP1 and melanin.

Therefore, we further addressed that elevated SASH1 increases the matrix protein levels in melanosomes. Western blotting demonstrated that SASH1 mutations significantly increased TYRP1, Rab 27a, Pmel17 and tyrosinase protein levels in SK‐MEL‐28 cells, a pigmented melanoma cell line and NHEMs (Fig. 7A and B). QRT‐PCR revealed that mutations of SASH1 up‐regulated Pmel17, TYRP1 and Rab 27a in SK‐MEL‐28 stable cells (Fig. 7C). IHC analysis also demonstrated that the expression of Pmel17, TYRP1 and Rab 27a was heterogeneously distributed in the epithelial cells in the tissues of DUH‐affected individuals (Fig. 7D and E). High levels of Pmel17, TYRP1 and Rab 27a were found in some hyperpigmentation regions in the affected epithelial layers of the Y551D‐affected individuals. In the hyperpigmentation plaque, the excessive production and secretion of melanin were obviously evident in the basal layers as well as in the suprabasal layers of the affected epidermal as (Fig. 7D).

## Discussion

We demonstrate that the reciprocal induction of p53 and SASH1 promotes cutaneous pigmentation by an autoregulatory p53/POMC/α‐MSH/Gαs/SASH1 loop in the skin. The SASH1‐mediated inducements of p53 and POMC were enhanced by SASH1 mutations. POMC had been reported to be induced by p53 overexpression and resulted in UV‐dependent hyperpigmentation or UV‐independent pathological hyperpigmentation 1. Hence, SASH1 mutations‐mediated‐POMC up‐regulation causes the pathological hyperpigmentations of DUH‐affected individuals. These data suggest that SASH1 activation in melanocytes represents a ‘UV sensor/effector’ for skin pigmentation or SASH1 mutation‐mediated up‐regulation is the ‘crime culprit’ of pathological hyperpigmentation of DUH, and its key mechanistic role is reciprocal inducement between SASH1 and p53. The identification of a positive feedback p53/POMC/α‐MSH/Gαs/SASH1 loop helps to indicate a key link in the p53 pathway and MC1R pathway by SASH1.

The transcriptional network of p53‐responsive genes produces proteins that interact with a large number of other signal transduction pathways in the cell and a number of positive and negative autoregulatory feedback loops act upon the p53 response. Feedback loops of p53‐ and p53‐responsive genes provide a means to connect the p53 pathway with other signal transduction pathways and co‐ordinate the cellular signals for growth and division 25. In this study, our findings suggest that the p53/POMC/α‐MSH pathway and MC1R/Gαs/cAMP/PKA cascade are connected by the SASH1 to form a autoregulatory p53/POMC/Gαs/SASH1 circuit to mediate the melanogenesis process.

Most recently, SASH1 is showed to be involved in autosomal‐dominant lentiginous 26 and autosomal‐recessive SASH1 variants (c.1849G>A; p.Glu617Lys) which are associated with a new genodermatosis with a pigmentation defects, palmoplantar keratoderma and skin carcinoma, and SASH1 is firstly reported to be predisposed to skin cancer 27. DUH is a clinically heterogeneous disorder that presents as generalized mottled pigmentation and was first reported by Ichikawa and Hiraga in 1933. Stuhrmann and colleagues identified the first locus responsible for autosomal‐recessive DUH, and this finding is consistent with recent evidence demonstrating that DSH and DUH are genetically distinct disorders 28. Zhang *et al*. mapped the causative gene of DSH to 1q11‐1q21 and found that a novel mutation of a heterozygous nucleotide A→G at position 2879 in exon 10 of the *DSRAD* gene is involved in DSH 29. Subsequent research on dyspigmentation has demonstrated that the pathogenic genetic variant that causes DSH is localized to the *DSRAD* gene on chromosome 1q 13, 30, 31, 32, 33, 34, 35. Expanding Stuhrmann and Nuber's findings and our own previous work providing photographic evidence of dyschromatosis presenting as large hyperpigmented bodies on DUH‐affected individuals 7, 28, 36, we believe that we have discovered the first locus associated with autosomal‐dominant DUH, identifying SASH1 as the causative gene of autosomal‐dominant DUH. This study suggests that SASH1 point mutations up‐regulate Pmel17, the protein that is responsible for melanin and melanin polymerization, and TYRP1, the protein that is responsible for melanin biosynthesis and tyrosinase stabilization *in vitro* and *in vivo*. The enhanced expression of TYRP, tyrosinase and Pmel17 is indicative of intensive activity of melanogenic enzymes in melanosomes or increased melanosome synthesis. Our data suggest that SASH1 mutations induce up‐regulation of Rab27a *in vitro* and *in vivo*, indicating that SASH1 mutations enhance the transport of melanosomes in melanocytes.

Taken above, our findings will enrich the communication of p53 pathway with other transduction pathways in cells and redefine the p53‐responsive genes and their associations, which will permit the perfections of the p53 programmed responses to stress and pathologic conditions.

## Conflict of interest

The authors confirm that there is no conflict of interest.

## Supporting information


**Figure S1** Endogenous SASH1 protein is unstable and mutation of *SASH1* induces the heterogeneous expression of SASH1 *in vitro*.

**Figure S2** Subcellular localization of SASH1.

**Figure S3** Exogenous p53 triggers expression of SASH1.

**Figure S4** p53 is not associated with SASH1 and SASH1 is not transcriptionally regulated by p53.

**Table S1** Proteins interacting with SASH1 were identified by MS analysis.
**Table S2** The peptide sequences of the SASH1 complex identified by SBP‐FLAG–SASH1 affinity purification.
**Table S3** Primers used for site directed mutagenesis, real time RT‐PCR and RNAi.
