# Supplementary figures and images for: A novel P53/POMC/Gαs/SASH1 autoregulatory feedback loop activates mutated SASH1 to cause pathologic hyperpigmentation
Source: J Cell Mol Med. 2016 Nov 25;21(4):802–15. doi: 10.1111/jcmm.13022 (PMC5345616; doi:10.1111/jcmm.13022)

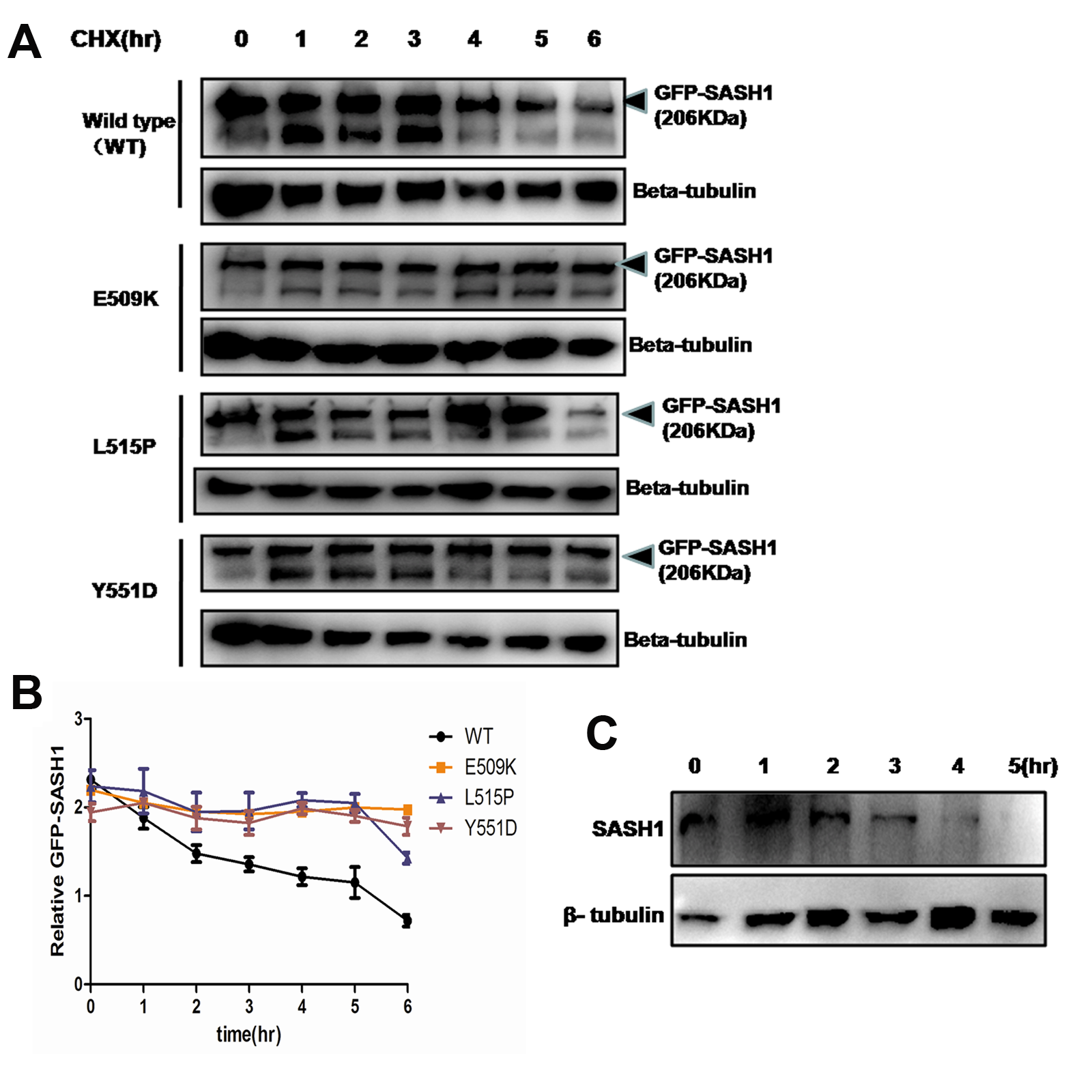

Supplement: Supplementary file 1 — Figure S1 Endogenous SASH1 protein is unstable and mutation of SASH1 induces the heterogeneous expression of SASH1 in vitro. [file JCMM-21-802-s001.tif]

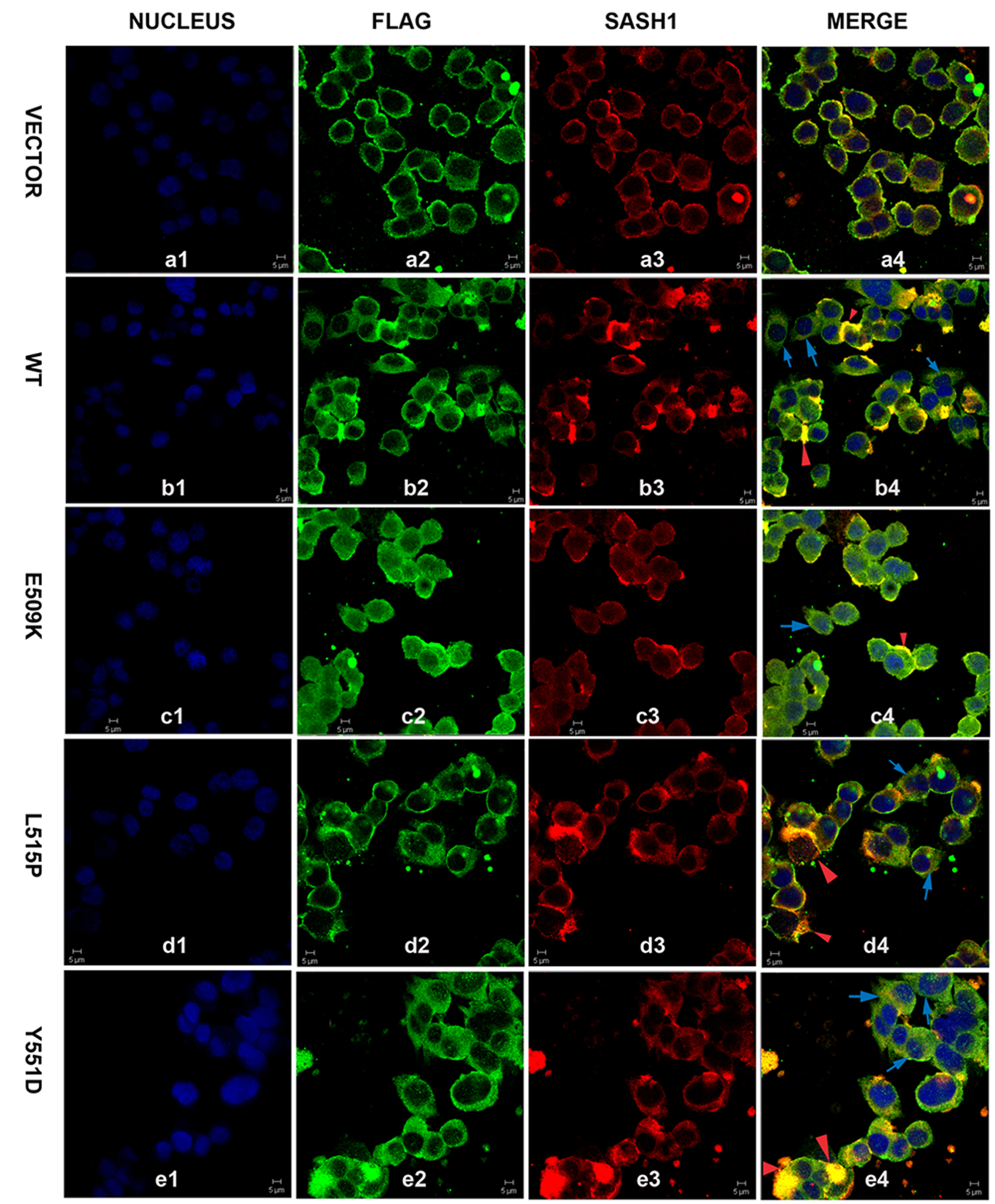

Supplement: Supplementary file 2 — Figure S2 Subcellular localization of SASH1. [file JCMM-21-802-s002.tif]

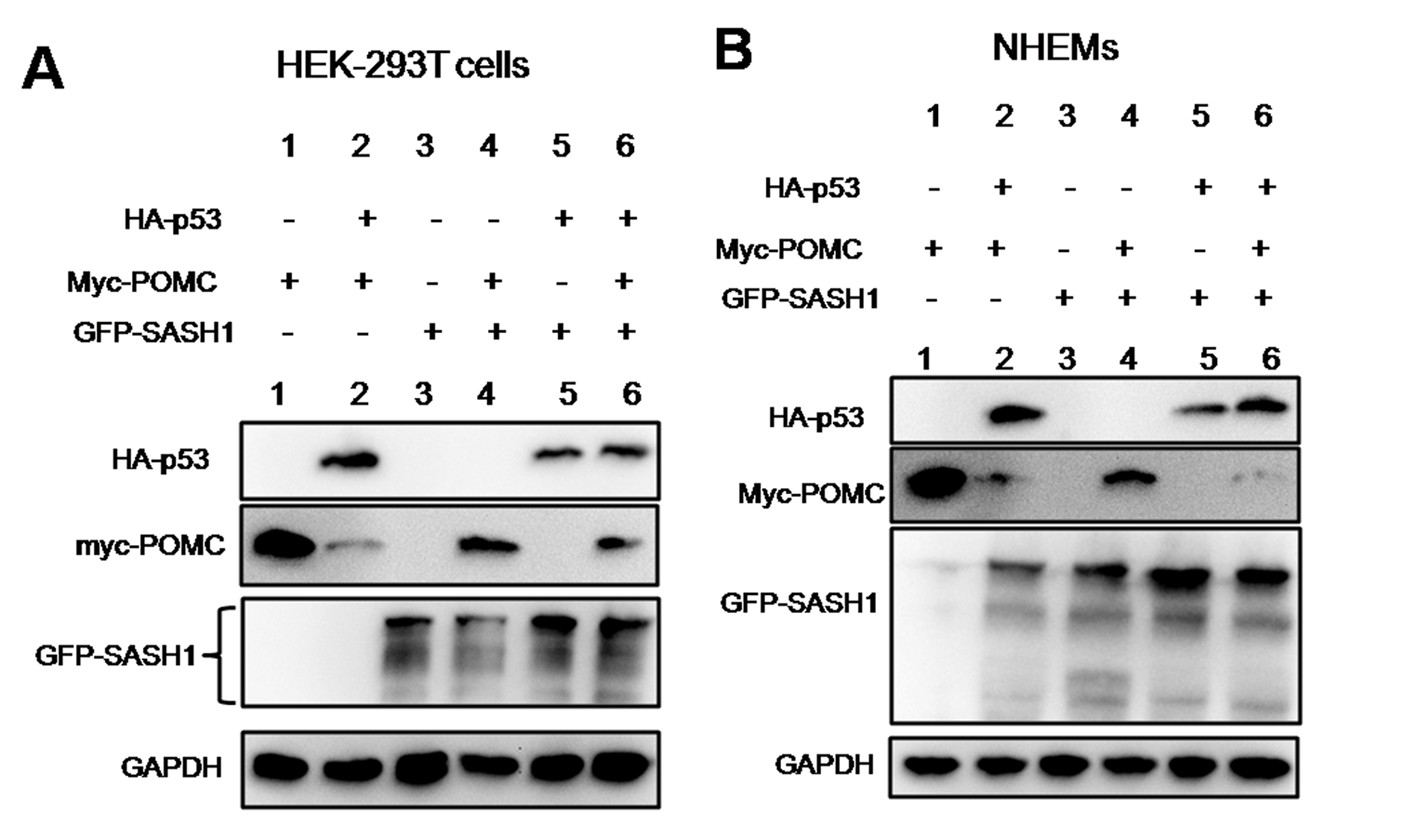

Supplement: Supplementary file 3 — Figure S3 Exogenous p53 triggers expression of SASH1. [file JCMM-21-802-s003.tif]

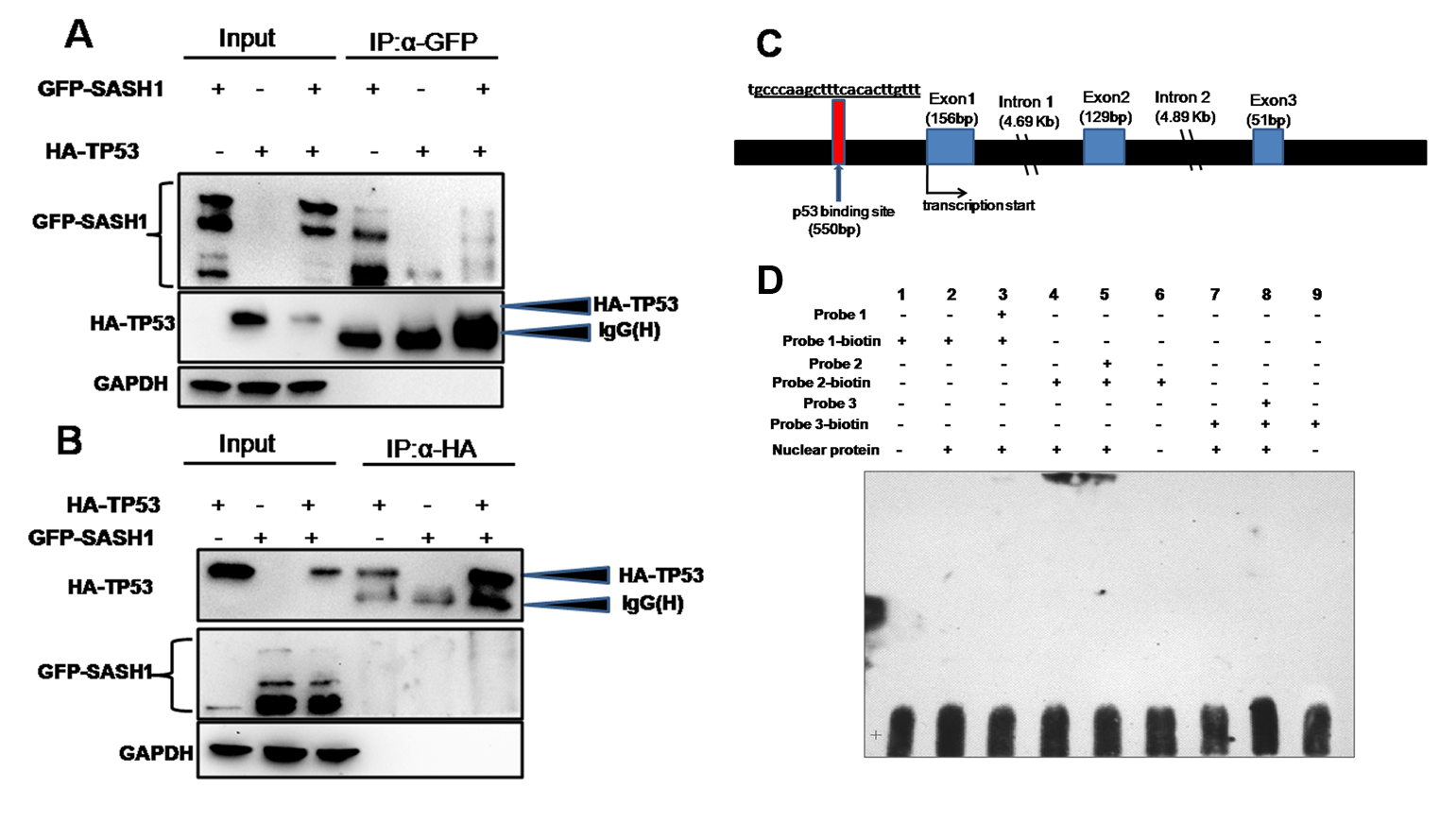

Supplement: Supplementary file 4 — Figure S4 p53 is not associated with SASH1 and SASH1 is not transcriptionally regulated by p53. [file JCMM-21-802-s004.tif]
