# Supplementary material for: A novel P53/POMC/Gαs/SASH1 autoregulatory feedback loop activates mutated SASH1 to cause pathologic hyperpigmentation
Source: J Cell Mol Med. 2016 Nov 25;21(4):802–15. doi: 10.1111/jcmm.13022 (PMC5345616; doi:10.1111/jcmm.13022)
Supplement: Supplementary file 5 — Table S1 Proteins interacting with SASH1 were identified by MS analysis. Table S2 The peptide sequences of the SASH1 complex identified by SBP‐FLAG–SASH1 affinity purification. Table S3 Primers used for site directed mutagenesis, real time RT‐PCR and RNAi. [file JCMM-21-802-s005.docx]

**Tables and Table Legends**

**Table S1.**  Proteins interacting with SASH1 were identified by MS analysis. Affinity-purified proteins were identified by MS analysis and the detailed peptide sequences are summarized in Tables S2

| PROTEIN name | Score | protein possibility | Total peptide | Unique peptide |
| --- | --- | --- | --- | --- |
| SASH1 | 200.3 | 1 | 37 | 20 |
| Gαs | 20.2 | 1 | 8 | 5 |
| CALM1 | 10.2 | 1 | 7 | 3 |

**Table S2. The peptide sequences of the SASH1 complex identified by SBP-FLAG–SASH1 affinity purification**

The precipitated proteins from stable A375 cells expressing SBP-FLAG–SASH1 were digested with trypsin. The supernatant was collected, dried and dissolved in 10% (v/v) acetonitrile and 0.8% formic acid solution. The peptides were analysed by LC-MS/MS.

| Protein name | Peptide sequence |
| --- | --- |
| SASH1(O94885) | K.KPSTEGGEEHVFENSPVLDERS  R.AVLLTAVELLQEYDSNSDQSGSQEKL  K.GEDVGYVASEITMSDEERI  R.VSQDLEVEKPDASPTSLQLR.S  R.VHTDFTPSPYDTDSLKI  K.LLEEEDLDELNIRD  K.LHAEGIDLTEEPYSDKH  K.PGAGTSEAFSR.L  KPLFFDGSPEKPPEDDSDSLTTSPSSSSLDTWGAG  K.MGTFFSYPEEEKA  KMITIEEALARL  RSLHVGSNNSDPMGKE  SLHVGSNNSDPMGK  ITIEEALAR  MITIEEALARL  RGVDLETLTENKL  IPSQPPPVPAK  TIEEALAR  KYFWQNFRK  SALYSGVHK |
| Gαs (P63092) | EAIETIVAAMSNLVPPVELANPENQFR  YTTPEDATPEPGEDPR  IEDYFPEFAR  MFDVGGQR  VLTSGIFETK |
| CALM1(P62158) | R.EADIDGDGQVNYEEFVQMMTAK |
|  |  |

**Table S3. Primers used for site directed mutagenesis ,real time RT-PCR and RNAi**

| Gene Name | Forward primer(5'-3') | Reverse primer(5'-3') |
| --- | --- | --- |
| Primers of site directed mutagenesis for E509K and L515P | ctgtagaaagtcttcacagttctcccagtgggcagagctc | gagctctgcccactgggagaactgtgaagactttctacag |
| Primers of site directed mutagenesis for Y551D | tgacgaagagccgcctgaccgaggccc | gggcctcggtcaggcggctcttcgtca |
| SASH1 | CGGGAAACGTCAAGTCGGA | ATCTCCTTTCTTGAGCTTGAG |
| TYRP1 | CACAGGCACAGGTACCACCTC | CTGAACTACCCTAGGTCTTCGTT |
| Pmel17 | AAGGTCCAGATGCCAGCTCAA | CTTTCACGGCTCTAGGACGTC |
| Rab 27a | AACTAGTGCTGCCAATGGGACA | TTTGATCGCACCACTCCTTC |
| Gαs | GTCCTTGCTGGGAAATCG | CGCAGGTGAAATGAGGGTAG |
| p53 | CCACCATCCACTACAACTACAT | TCCCAGCACAGGCACAAA |
| POMC | AGTTCAAGAGGGAGCTGACTGG | CATGAAACCGCCGTAGCG |
| GAPDH | CACCCACTCCTCCACC TTTG | ACCACCCTGTTGCT GTAGCC |
| Gαs siRNA 1 | GAGGACUACUUUCCAGAAUTT | AUUCUGGAAAGUAGUCCUCTT |
| Gαs siRNA 2 | GCAGCUACAACAUGGUCAUTT | AUGACCAUGUUGUAGCUGCTT |
| POMC siRNA1 | ACCUCACCACGGAAAGCAATT | UUGCUUUCCGUGGUGAGGUTT |
| POMC siRNA2 | AGUACGUCAUGGGCCACUUTT | AAGUGGCCCAUGACGUACUTT |
| GAPDH | GUAUGACAACAGCCUCAAGTT | CUUGAGG CUGUUGUCAUACTT |
| Negative Control | UUCUUCGAACGUGUCACGUTT | ACGUGACACGUUCGG AGAATT |
