# Supplementary material for: A novel P53/POMC/Gαs/SASH1 autoregulatory feedback loop activates mutated SASH1 to cause pathologic hyperpigmentation
Source: J Cell Mol Med. 2016 Nov 25;21(4):802–15. doi: 10.1111/jcmm.13022 (PMC5345616; doi:10.1111/jcmm.13022)
Supplement: Supplementary file 6 [file JCMM-21-802-s006.docx]

**Supplementary Figures**

**Fig. S1 Endogenous SASH1 protein is unstable and mutation of *SASH1* induces the heterogeneous expression of SASH1 *in vitro***

(**A**) Mutant SASH1 proteins are more stable than the wild type SASH1 protein. Stable HEK-293T cells were treated with CHX (20 μg/ml) for the indicated times and analyzed by western blotting. The amount of SASH was quantified by densitometry and normalized to beta-tubulin. CHX resulted in the degradation of wild type SASH1 protein, which had a half-life of 4 hr. Under a 6-hr or longer treatment with CHX, CHX began to induce the degradation of mutant SASH1 proteins. (**B**) The intensity of GFP-SASH1 was quantified by densitometry and normalized to β-tubulin (n=3). (**c**)Endogenous SASH1 is an unstable protein. HEK-293T cells were deprived of FBS for the indicated time and lysed and subjected to western blot to detect the endogenous SASH1 levels.

**Fig. S2 Subcellular localization of SASH1**

The fluorescence signals that were detected by confocal microscopy indicate that the over-expression or mutation of SASH1 results in the heterogeneous expression of SASH1 *in vitro* in A375 stable cells. The green fluorescence represents the Flag-tag label. Both exogenous and endogenous SASH1 are labeled with a red fluorescent tag. The nuclei are labeled with DAPI (in blue). The yellow fluorescence indicates the overlap of the green and red fluorescent staining. The red arrowheads indicate the activated SASH1-Flag fusion proteins that were expressed in the cytoplasm of WT-A375 cells or mutant-A375 cells. The blue arrowheads indicate the regions that do not express the activated SASH1-Flag fusion protein in the cytoplasm of WT-A375 or mutant-A375 cells. The endogenous SASH1 presents a uniform pattern of expression in all of the VECTOR-A375 cells (Figure 3A-a4). Bar=5 μm.

**Fig. S3** Exogenous p53 triggers expression of SASH1

**(A)** Exogenous p53 caused up-regulation of exogenous SASH1 in HEK-293T cells. HA-TP53, GFP- SASH1 and myc-POMC were transfected into HEK-293T cells for transient expression. Cells were lysed in 0.5% NP40 buffer containing protease inhibitors and subjected to western blot along with GAPDH as loading control. **(B)** Exogenous p53 caused up-regulation of exogenous SASH1 in NHEMs.

**Fig. S4** p53 is not associated with SASH1 and SASH1 is not transcriptionally regulated by p53

**(A) and (B)** HEK-293T cells were co-transfected with the pEGFP-C3-SASH1 and Pcdna 3.0-HA-p53 vectors. At 24 hr post-transfection, GFP-SASH1 was immunoprecipitated, and the associated HA-p53 was detected by western blot analysis using an anti-HA antibody. Similarly, HA-p53 was immunoprecipitated, and the associated GFP-SASH was detected by western blot analysis using an anti-GFP antibody. **(C)** shows a schematic representation of the SASH1 locus, which indicates location of a p53-binding consensus sequence . **(D)** EMSA analyses demonstrated that there was none of among three probes of SASH1 gene promoter to bind p53 recombined protein.
